# Quantitative study of the effects of early standardized ambulation on sleep quality in patients after hepatectomy

**DOI:** 10.3389/fsurg.2022.941158

**Published:** 2022-09-23

**Authors:** Chun-yan Ni, Guo-jun Hou, Ya-yuan Tang, Jing-jing Wang, Wen-jun Chen, Yuan Yang, Zhi-hong Wang, Wei-ping Zhou

**Affiliations:** ^1^ Eastern Hepatobiliary Surgery Hospital, Second Military Medical University, Shanghai, China; ^2^ Suzhou Science / Technology Town Hospital, Suzhou, China

**Keywords:** liver resection, physical activity level, sleep, enhanced recovery after surgery, nursing

## Abstract

**Background:**

Sleep quality has been always an important problem for patients after hepatectomy. The main purpose of the study is to investigate the effects of early ambulation on sleep quality in patients after liver resection *via* a quantitative study.

**Methods:**

Patients undergoing liver tumor resection were randomly divided into two groups, and the Pittsburgh Sleep Quality Index (PSQI) was used to assess the postoperative activities and sleep quality.

**Results:**

Patients who started early ambulation after liver resection had significantly better sleep quality, faster recovery of gastrointestinal function and shorter lengths of postoperative hospital stay compared with the control group. And there was no significant difference in the incidence of postoperative complications between the two groups.

**Conclusion:**

Early standardized physical activities are feasible for patients after liver resection, which can significantly improve patient's sleep quality, reduce patient's pain and the nursing workload, and achieve rapid recovery.

## Introduction

Partial hepatectomy still remains the most commonly used curative treatment for liver tumor. However, clinical observations have shown that most patients with liver malignancies also have symptoms such as insomnia, emotional instability, lethargy and fatigue. Sleep is a basic physiological process of the human body, an important part of body recovery and integration and consolidation of memory and an indispensable part of health. People with poor sleep quality often show mood depression, nervousness, irritability, anxiety and anergia. Poor sleep quality tends to cause or aggravate the patient's psychosomatic symptoms, which in turn can worsen sleep quality, forming a vicious cycle (1–5). Studies have shown that lack of sleep can prolong the length of hospital stay, increase the chance of infection and even increase the mortality rate (6–8). Accurate care under the concept of enhanced recovery after surgery (ERAS) can not only relieve the fear and anxiety of patients but also reduce the degree of postoperative pain as well as the use and side effects of analgesics, thereby improving sleep quality and promoting recovery from a disease (9).

Early ambulation has become an indispensable part of postoperative care. It can reduce muscle loss and complications including pneumonia and deep vein thrombosis (10–12). The “Chinese expert consensus on enhanced recovery after hepatectomy (2017)” and expert consensuses from other sources clearly state that early activities refer to activities with reasonable plans and goals and recommended that daily activity goals after surgery should be established and that the amount of activity should be increased each day (13). However, no specific quantitative plan was included in these consensuses. In this regard, our research team conducted a series of studies that showed that early ambulation starting the next day after surgery is safe and feasible (10). Patients who started early ambulation had longer postoperative sleep duration than those in the control group. However, in our previous studies, the effects of early standardized ambulation on sleep quality in patients after liver resection were not quantified. Therefore, in the present study, we further investigated the relationship between the early ambulation and sleep quality after surgery using a wireless smart wristband, the Fitbit Flex2 (14, 15), and the Pittsburgh Sleep Quality Index (PSQI) (4, 16) to monitor in real time, assess and correctly understand the postoperative physical activity level and sleep quality of patients, thereby improving the compliance and effectiveness of activities and thus promoting the early recovery of patients.

## Materials and methods

### General information

#### Sample size and power analysis

This study was approved by the clinical research ethics committee of the Eastern Hepatobiliary Surgery Hospital (Shanghai, China). Written informed consent was obtained from each patient according to the policies of the committee. From March 2018 to July 2018, open liver resection was performed at the Third Department of Hepatic Surgery, Eastern Hepatobiliary Surgery Hospital in 312 patients. According to the published report, assuming a type-I error of 5% (*α *= 0.05), a power of 80% for a 2-tailed log-rank test (*β *= 0.2), and about 10% post-randomization dropout, the sample size was 120 patients, with 60 patients in each group.

#### Study design and randomisation technique

**Figure 1:**
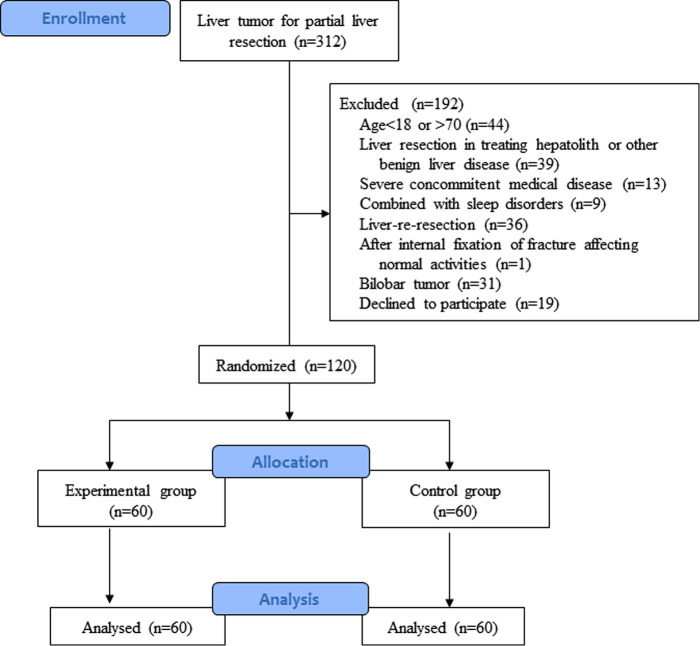
Flowchart of the patients enrolled in this study.

Based on the different protocols for postoperative activities, patients who met the inclusion criteria were randomly assigned to 2 groups, the control group and the experimental group (early standardized activity group), with 60 patients in each group (Figure 1). Randomization was done by a nurse who was not involved in this study when the patients were assessed suitable to be included into this study, and the randomization process was computer generated. There were no significant differences in age, sex, tumor types, extent of resection or surgical approach between the two groups (Table 1). The study was approved by the Institutional Review Board of the Eastern Hepatobiliary Hospital. Patients' decision to participate in the study was voluntary, and informed consent was obtained for randomization and treatment. This project has been registered in the Chinese Clinical Trial Registry (registration number: ChiCTR-TRC-13003270).

**Table 1:** Comparison of the general data between the two groups of patients.

	Control group (*n* = 60)	Experimental group (*n* = 60)	Test value		*P*
			*t*	*χ* ^2^	
Age	53.4 ± 13.6	57.2 ± 18.4	1.29		0.20
Sex (M/F)	53/7	54/6		0.09	0.77
Tumor size	7.6 ± 4.8	6.1 ± 4.7	1.73		0.09
HBV (+)	59	57		0.47	0.49
Presence of cirrhosis	52	50		0.26	0.61
**Tumor types**					
Malignant tumor	58	55		1.37	0.24
Benign tumor	2	5			
**Surgical procedure**					
Hemihepatectomy	28	24		0.54	0.46
Local tumor resection	32	36			
Operative time (min)	142.2 ± 47.8	133.9 ± 60.8	0.83		0.41
Liver warm ischemia time (min)	17.3 ± 10.2	16.6 ± 9.7	0.39		0.70
Intraoperative blood loss (ml)	238.0 ± 209.7	256.8 ± 221.4	0.48		0.63
Intraoperative blood transfusion (cases)	7	6		0.09	0.77
Intraoperative blood transfusion amount (units)	2.5 ± 1.1	3.3 ± 2.1	1.89		0.06

### Inclusion and exclusion criteria

The inclusion criteria were as follows: Patients who underwent first surgery for liver tumor, were aged from 18 to 70 years and had no movement disorder; The preoperative examination indicated no severe cardiopulmonary dysfunction, history of psychosis, sleep disorders or cognitive dysfunction.

The exclusion criteria were as follows: ① patients with related bone joint diseases affecting normal activities; ② patients with brain disorders or mental disorders that prevented them from cooperating; ③ patients with sleep disorders before surgery.

### Protocol for physical activities in the control group

For the control group, postoperative activities were performed following the guidance of the postoperative nursing routine after liver surgery in the Handbook of Nursing in Hepato-Pancreato-Biliary Surgery (17). The detailed protocol is as follows: patients started physical activities on the bed within 2 days after surgery; patients started to exercise while standing on day 3 after surgery; patients started ambulation on day 4 and day 5 after surgery. The assessment criteria were as follows: the patients expressed a feeling of tolerance; no symptoms including palpitation, dizziness, shortness of breath and pain in the wound were reported after the activity; no obviously increased heart rate, elevated blood pressure or extreme fatigue was present after the activity.

### Protocol for physical activities in the early standardized activity group

A postoperative safety assessment was completed to exclude active bleeding. Patients and their families were informed about the detailed postoperative activity plan before surgery. After surgery, the nurse-in-charge used a form to indicate the level of daily activities, and the form was sent to the patients and their families at 8:00 am. At completion of activities, the family members would place a check mark on the form, which improved the participation of family members. To enhance safety and effectiveness, the amount of activity was allocated for three time periods (morning, noon and evening). The nurse-in-charge and the night-shift nurses supervised patients who had not completed the minimally required activity level as they completed the assigned activity. The protocol of activities during the early stage is described as follows. (1) On the day of surgery, passive movement of the arms and legs, including the ankles, on the bed was major form of activity. (2) On the first day after surgery, patients were allowed to sit on the bed with their legs hanging down over the edge of the bed. (3) On the second day after surgery, the secured drainage tube was changed or removed. Following the “trilogy of getting up” (siting on the bed for 1 min, siting with legs hanging down over the edge of the bed and standing at the bedside for 1 min), patients were allowed to ambulate outside the ward 2 to 3 times, with a total walking distance of 250 to 500 m. (4) From the third day after surgery, patients were enforced to ambulate outside the ward 5 times, with a total walking distance of greater than 1500 m. According to the patient's condition, pain assessment was performed promptly. The amount of activity was eventually increased based on the patient's vital signs and tolerance.

### Measurement of physical activity and sleep

From the day of surgery, both groups of patients were asked to wear a Fitbit Flex2, a wireless smart wristband, for step counts and monitoring of walking distance and sleep time. The physician-in-charge or nurse-in-charge synchronized the wristband information to the corresponding account through a mobile phone or computer using Bluetooth technology at least 2 times a day and observed the outcomes of the activities. For patients in the early standardized activity group, those who did not complete the minimum activity distance were asked to complete the activity under supervision. The daily activity and sleep time of the patients were recorded over time. Meanwhile, patients were encouraged to download the Fitbit App to monitor their activities in real time. In the control group, the activity was determined according to the degree of tolerance of the liver surgical care routine.

### Sleep quality assessment

The sleep quality at 1 week before surgery and 1 week after surgery were compared in the two groups using the PSQI. The PSQI measures 7 different factors of sleep including sleep quality, sleep onset latency, sleep duration, sleep efficiency, sleep disorders, use of sleeping medication and daytime dysfunction. Each factor is scored on a scale of 0–3. The overall score ranges from 0 to 21, and a higher score denotes poorer sleep quality of a patient. The questionnaire was distributed by the nurses 1 day before the surgery and 5 days after the surgery. The patients completed the questionnaire by themselves. A total of 240 copies were distributed and collected, with an effective recovery rate of 100%.

### Indicators of observation

(1) The postoperative walking distance, the number of paces, the pain score, the sleeping time, and PSQI score were recorded. (2) Postoperative gastrointestinal function recovery was assessed based on whether nausea, vomiting, bloating, and/or diarrhoea occurred; the time of first exhaust and flatus were recorded. (3) The postoperative complications and the length of the postoperative hospital stay were recorded. Criteria for patients to be discharged home in both groups followed the recommendations by Dr R. M. van Dam (18), which included normal or decreasing serum bilirubin, good pain control with oral analgesics only, tolerance of solid food, no intravenous fluids, independent patient mobility or at the preoperative level, and patient's willingness to go home.

### Statistical analysis

Statistical analysis was performed based on an intention-to-treat analysis and was performed using the IBM SPSS Statistics (Version 19.0. Armonk, NY: IBM Corp) package. Quantitative data are expressed as the means ± standard deviation. A t-test or Mann-Whitney test was used to compare continuous variables such as the pain scores, walking distance, sleep duration, PSQI scores, first exhaust and defecation time. The chi-square test or Fisher's exact probability method was used to compare discrete variables such as the incidence of complications and gastrointestinal function. A value of *P* < 0.05 was considered statistically significant.

## Results

The demographic data of the two groups are shown in Table 1. There were no significant differences in in age, sex, tumor types, resection scope, and operative details (*P* > 0.05)

Based on the protocol of the early standardized activity group, the number of paces and walking distance were much higher in the early standardized activity group than in the control patients. Pain scores, walking distance, step count, sleep duration and number of sleep interruptions were used to evaluate the postoperative recovery. From the 4th day after hepatectomy, the pain scores in the early standardized activity group were significantly lower than those in the control group. In addition, sleeping times were longer with less sleep interruptions than those in the control group in the control group (*P* < 0.05, Table 2).

**Table 2:** Postoperative walking distance, pain scores and sleep quality in the two groups.

	Walking distance (m)	Step count	Pain score	Sleep duration (h)	Number of sleep interruptions
**Control group**					
Day 1 after surgery	0	0		5.4 ± 3.9	5.1 ± 4.2
Day 2 after surgery	13.0 (0–110)	35.0 (0–311)	3.0 ± 2.7	6.5 ± 2.7	7.9 ± 5.8
Day 3 after surgery	101.2 ± 70.3	272.3 ± 149.3	2.8 ± 1.9	7.1 ± 2.1	5.4 ± 3.9
Day 4 after surgery	457.8 ± 282.1	909.2 ± 418.0	3.5 ± 2.1	7.0 ± 2.3	4.5 ± 2.6
Day 5 after surgery	1210.1 ± 742.2	3765.2 ± 209.9	3.2 ± 2.2	6.2 ± 4.3	4.2 ± 3.7
**Experimental group**					
Day 1 after surgery	0 (0–50)	0 (0–240)		5.2 ± 4.1	5.2 ± 3.1
Day 2 after surgery	310 (220–550)*	898 (550–1210)*	3.1 ± 2.6	6.7 ± 2.9	4.0 ± 1.6*
Day 3 after surgery	1551.3 ± 240.9*	3887.2 ± 891.9*	3.0 ± 1.9	7.9 ± 1.8**	3.6 ± 2.1*
Day 4 after surgery	1677.7 ± 321.2*	4022.9 ± 929.1*	2.8 ± 1.8**	7.3 ± 2.6	3.2 ± 1.7*
Day 5 after surgery	1638.2 ± 283.7*	4189.6 ± 901.4*	2.5 ± 1.9**	7.8 ± 3.1**	3.1 ± 1.2**

*P < 0.01.

**P < 0.05.

Using the “discharge criteria of ERAS” for the CS group of patients, the postoperative hospital stay for the early standardized activity group would have been significantly shorter than that of the control group (*P* = 0.04) (Table 3). The duration of time the patients had nausea/vomiting and the time until first passed flatus and first exhaust following surgery were significantly shorter in the early standardized activity group than in the control group (Table 3). In addition, there was no perioperative mortality, reoperation in this study and no significant differences in the postoperative complications between the two groups.

**Table 3:** Postoperative gastrointestinal function and length of hospital stay in the two groups.

	Control group (*n* = 60)	Experimental group (*n* = 60)	Test value		*P*
			*t*	*χ* ^2^	
**Postoperative gastrointestinal function**					
Nausea/vomiting	9	3		2.13	0.14
Abdominal distention	10	2		4.29	0.04
Diarrhea	5	3		0.10	0.75
First exhaust time (days)	3.3 ± 2.2	2.1 ± 1.7	3.31		<0.01
First defecation time (days)	3.2 ± 2.4	2.3 ± 2.0	2.21		0.03
Length of postoperative hospital stay (days)	7.6 ± 2.2	6.7 ± 2.4	2.12		0.04

This study showed no statistically significant difference in sleep quality between the two groups before operation, but the exception of a statistically significant difference in sleep quality on day 5 after surgery (*P* < 0.05). PSQI assessment was performed in both groups after surgery. The sleep quality, sleep latency, sleep disturbance, use of sleeping medication, daytime function and total PSQI scores all showed statistically significant differences (*P* < 0.05) (Tables 4, 5).

**Table 4:** Comparison of sleep quality assessment (PSQI) in the two groups.

Group	*N*	On the day of admission	On day 5 after surgery	*t*	*P*
Experimental group	60	7.6 ± 2.2	9.6 ± 3.2	1.77	>0.05
Control group	60	7.8 ± 3.2	13.4 ± 5.8	8.09	<0.01
*t*		1.05	7.33		
*P*		>0.05	<0.01		

**Table 5:** Comparison of postoperative sleep quality assessment (PSQI) between the two groups.

Item	Control group (*n* = 60)	Experimental group (*n* = 60)	Test value	*P*
Sleep quality	2.52 center1.70	1.70 center1.21	3.633	<0.01
Sleep latency	2.01 center1.81	1.72 center1.08	4.216	<0.01
Sleep duration	1.70 center0.99	1.44 center0.70	1.543	0.165
Sleep efficiency	1.22 center0.92	1.01 center0.51	1.899	0.063
Sleep disorder	1.56 center0.62	1.07 center0.54	3.626	<0.01
Use of sleeping medication	1.57 center0.61	0.98 center0.52	4.233	<0.01
Daytime function	2.02 center1.92	1.56 center0.68	3.986	<0.01
Total PSQI score	13.4 center5.8	9.6 center3.2	7.866	<0.01

## Discussion

### Analysis of sleep quality in patients after liver resection

In this study the total PSQI scores, the sleep quality, sleep latency, sleep disturbance, use of sleeping medication, and daytime function all showed statistically significant differences (*P* < 0.05) between the two groups. The possible reasons may be related to postoperative pain, postural discomfort, irritation due to indwelling catheters, anxiety, nervousness and the ward environment, all of which can affect the patient's sleep quality. However, postoperative pain, irritation due to indwelling catheters and postural discomfort were the main factors that affected postoperative sleep quality. Therefore, according to the concept of ERAS, continuous analgesia, early removal of the drainage tube and early feeding and ambulation are the most important methods to alleviate sleep disorders (9, 18, 19).

### Wireless smart wristband measurement assessed the activity level and sleep duration

The Fitbit Flex2 wireless smart wristband was used to facilitate real-time monitoring of step counts, walking distance and sleep duration in adult patients in this study. The results showed that the walking distance, step counts and number of sleep interruptions were significantly different (*P* < 0.05) between the experimental and control groups from day 2 to 5. Real-time monitoring by the wristband showed that sleep disorders were generally present in postoperative patients. The main manifestations included difficulty in initiating asleep (sleep latency >30 min), poor sleep quality with many nightmares and difficulty in maintaining sleep (awakening more than 3 times per night and insomnia after awakening in the early morning). The total sleep duration was less than 6 h. On the second day, the patients felt dizzy and exhibited a lack of energy, lethargy and general malaise. Patients and their families were encouraged to make full use of Fitbit's real-time monitoring to participate in patient care as a supervisory alliance (20, 21). The patient's postoperative activity and time were quantified, and the staff of the 3 shifts supervised the progress daily. The nurse manager, physician-in-charge and nurse-in-charge performed the overall assessments and moderately adjusted the protocol if needed (22–25).

### Early ambulation after liver resection contributed to rapid recovery

In our previous studies, early forced ambulation was proved to be safe and feasible without increasing the incidence of postoperative complications. With concurrent use of multimodal analgesia, early postoperative activities can significantly improve intestinal paralysis, promote the recovery of various organs and nutrient absorption, reduce and improve the symptoms of abdominal distension and abdominal pain, improve organ autonomic function, reduce the activity of sympathetic nerves of digestive organs and improve the excitability of parasympathetic nerves to enhance gastrointestinal motility (9, 10, 26). However, sleep quality was not a main endpoint in previous report.

Further to investigative the importance of sleep quality after surgery, PSQI was used to score and compare the physical activities and sleep quality between the two groups. Early standardized physical activities can significantly improve patient's sleep quality, reduce patient's pain. As a result, the length of the postoperative hospital stay was also significantly reduced (*P* < 0.05). Although there was no statistically significant difference in the incidence of postoperative complications between the groups. Studies suggest that early activities can significantly prolong nighttime sleep and improve sleep quality, which can reduce anxiety symptoms and pain and enhance self-confidence and well-being after recovery. Moreover, early activities can reduce daytime sleep time and increase nighttime sleep time to improve sleep quality, indirectly promoting postoperative recovery (6, 10, 27).

### Standardized activities can help improve sleep quality in patients after liver resection

Sleep is an important physiological phenomenon, and sleep quality is the assessment of how well one sleeps. Good sleep can promote recovery from disease and strengthen the body's physiological functions. Due to the disease itself and changes in the external environment, sleep can be seriously affected in hospitalized patients (28, 29). Patients undergoing surgery are considered a specific population who require more high-quality sleep. However, their sleep is often affected by many factors. The present study has shown that patients have poor sleep quality due to surgery, but standardized early activities can help significantly improve their sleep quality. Therefore, sleep care should be emphasized in postoperative patients, and a standardized sleep care model should be established (30, 31). A standardized assessment tool should be used to measure relevant factors, help perform prospective intervention and develop a personalized care plan for individuals to improve sleep (32–38). Methods to improve sleep quality should be considered as a basic part of health education, and patients should be educated about factors that impact sleep to improve sleep quality and comfort.

## Conclusion

In conclusion, this study showed that early standardized physical activities were feasible for patients after liver resection. They could significantly increase the patient's sleep duration and improve the sleep quality to reduce the patient's pain, the length of hospital stays, the nursing workload and thus achieve rapid recovery.

## Data Availability

The original contributions presented in the study are included in the article/Supplementary Material, further inquiries can be directed to the corresponding author/s.

## References

[1] Ohayon M Wickwire EM Hirshkowitz M Albert SM Avidan A Daly FJ National sleep foundation's sleep quality recommendations: first report. Sleep Health. (2017) 3(1):6–19. 10.1016/j.sleh.2016.11.006 28346153

[2] Fontes F Goncalves M Maia S Pereira S Severo M Lunet N. Reliability and validity of the Pittsburgh sleep quality index in breast cancer patients. Support Care Cancer. (2017) 25(10):3059–66. 10.1007/s00520-017-3713-9 28455545

[3] Beck SL Schwartz AL Towsley G Dudley W Barsevick A. Psychometric evaluation of the Pittsburgh sleep quality index in cancer patients. J Pain Symptom Manage. (2004) 27(2):140–8. 10.1016/j.jpainsymman.2003.12.002 15157038

[4] Carpenter JS Andrykowski MA. Psychometric evaluation of the Pittsburgh sleep quality index. J Psychosom Res. (1998) 45(1):5–13. 10.1016/S0022-3999(97)00298-5 9720850

[5] Covarrubias-Gomez A Mendoza-Reyes JJ. Evaluation of sleep quality in subjects with chronic nononcologic pain. J Pain Palliat Care Pharmacother. (2013) 27(3):220–4. 10.3109/15360288.2013.816405 24004315

[6] Ju M Tao Y Lu Y Ding L Weng X Wang S Evaluation of sleep quality in adolescent patients with osteosarcoma using Pittsburgh sleep quality index. Eur J Cancer Care. (2019) 28(4):e13065. 10.1111/ecc.13065 31012535

[7] Silva GE An MW Goodwin JL Shahar E Redline S Resnick H Longitudinal evaluation of sleep-disordered breathing and sleep symptoms with change in quality of life: the sleep heart health study (SHHS). Sleep. (2009) 32(8):1049–57. 10.1093/sleep/32.8.1049 19725256 PMC2717195

[8] Tsai PS Wang SY Wang MY Su CT Yang TT Huang CJ Psychometric evaluation of the Chinese version of the Pittsburgh sleep quality index (CPSQI) in primary insomnia and control subjects. Qual Life Res. (2005) 14(8):1943–52. 10.1007/s11136-005-4346-x 16155782

[9] Ni CY Yang Y Chang YQ Cai H Xu B Yang F Fast-track surgery improves postoperative recovery in patients undergoing partial hepatectomy for primary liver cancer: a prospective randomized controlled trial. Eur J Surg Oncol. (2013) 39(6):542–7. 10.1016/j.ejso.2013.03.013 23562361

[10] Ni CY Wang ZH Huang ZP Zhou H Fu LJ Cai H Early enforced mobilization after liver resection: a prospective randomized controlled trial. Int J Surg. (2018) 54(Pt A):254–8. 10.1016/j.ijsu.2018.04.060 29753000

[11] Henriksen MG Hansen HV Hessov I. Early oral nutrition after elective colorectal surgery: influence of balanced analgesia and enforced mobilization. Nutrition. (2002) 18(3):263–7. 10.1016/S0899-9007(01)00749-3 11882401

[12] Henriksen MG Jensen MB Hansen HV Jespersen TW Hessov I. Enforced mobilization, early oral feeding, and balanced analgesia improve convalescence after colorectal surgery. Nutrition. (2002) 18(2):147–52. 10.1016/S0899-9007(01)00748-1 11844646

[13] Jia W Liu W Qiao X. Chinese expert consensus on enhanced recovery after hepatectomy (version 2017). Asian J Surg. (2019) 42(1):11–8. 10.1016/j.asjsur.2018.01.007 29627391

[14] Chu AH Ng SH Paknezhad M Gauterin A Koh D Brown MS Comparison of Wrist-Worn Fitbit Flex and Waist-Worn actigraph for measuring steps in free-living adults. PLoS One. (2017) 12(2):e0172535. 10.1371/journal.pone.0172535 28234953 PMC5325470

[15] Noah JA Spierer DK Gu J Bronner S. Comparison of steps and energy expenditure assessment in adults of fitbit tracker and ultra to the actical and indirect calorimetry. J Med Eng Technol. (2013) 37(7):456–62. 10.3109/03091902.2013.831135 24007317

[16] Hinz A Glaesmer H Brahler E Löffler M Engel C Enzenbach C Sleep quality in the general population: psychometric properties of the Pittsburgh sleep quality index, derived from a German community sample of 9284 people. Sleep Med. (2017) 30:57–63. 10.1016/j.sleep.2016.03.008 28215264

[17] Zhixia Y Caixia L Qin Z. Handbook of nursing in hepato-pancreato-biliary surgery. Shanghai: Shanghai Scientific and Technological Literature Press (2017).

[18] Paixao DL Poyares D de Paula MS Duarte JW Castelo PM Ambrogini-Júnior O Evaluation of home polysomnography findings, quality of sleep, and fatigue in inflammatory bowel disease: a case series. J Clin Sleep Med. (2019) 15(1):39–45. 10.5664/jcsm.7566 30621826 PMC6329559

[19] Albu S Umemura G Forner-Cordero A. Actigraphy-Based evaluation of sleep quality and physical activity in individuals with spinal cord injury. Spinal Cord Ser Cases. (2019) 5:7. 10.1038/s41394-019-0149-0 30675391 PMC6341092

[20] Liang Z Chapa-Martell MA. Accuracy of fitbit wristbands in measuring sleep stage transitions and the effect of user-specific factors. JMIR mHealth and uHealth. (2019) 7(6):e13384. 10.2196/13384 31172956 PMC6592508

[21] Cook JD Prairie ML Plante DT. Utility of the Fitbit Flex to evaluate sleep in major depressive disorder: a comparison against polysomnography and Wrist-Worn actigraphy. J Affect Disord. (2017) 217:299–305. 10.1016/j.jad.2017.04.030 28448949 PMC5509938

[22] Godino JG Wing D de Zambotti M Baker FC Bagot K Inkelis S Performance of a commercial multi-sensor wearable (fitbit charge HR) in measuring physical activity and sleep in healthy children. PLoS One. (2020) 15(9):e0237719. 10.1371/journal.pone.0237719 32886714 PMC7473549

[23] Liao Y Robertson MC Winne A Wu IHC Le TA Balachandran DD Investigating the within-person relationships between activity levels and sleep duration using fitbit data. Transl Behav Med. (2021) 11(2):619–24. 10.1093/tbm/ibaa071 32667039 PMC7963288

[24] Haghayegh S Khoshnevis S Smolensky MH Diller KR Castriotta RJ. Accuracy of wristband fitbit models in assessing sleep: systematic review and meta-analysis. J Med Internet Res. (2019) 21(11):e16273. 10.2196/16273 31778122 PMC6908975

[25] Baroni A Bruzzese JM Di Bartolo CA Shatkin JP. Fitbit flex: an unreliable device for longitudinal sleep measures in a non-clinical population. Sleep Breath. (2016) 20(2):853–4. 10.1007/s11325-015-1271-2 26449552

[26] Dubljanin-Raspopovic E Markovic-Denic L Ivkovic K Nedeljković U Tomanović S Kadija M The impact of postoperative pain on early ambulation after hip fracture. Acta Chir Iugosl. (2013) 60(1):61–4. 10.2298/ACI1301061D 24669564

[27] Kuo CE Liu YC Chang DW Young CP Shaw FZ Liang SF. Development and evaluation of a wearable device for sleep quality assessment. IEEE Trans Biomed Eng. (2017) 64(7):1547–57. 10.1109/TBME.2016.2612938 28113301

[28] Buyukyilmaz FE Sendir M Acaroglu R. Evaluation of night-time pain characteristics and quality of sleep in postoperative Turkish orthopedic patients. Clin Nurs Res. (2011) 20(3):326–42. 10.1177/1054773811406110 21521827

[29] Okkesim CE Serbest S Tiftikci U Çirpar M. Prospective evaluation of preoperative and postoperative sleep quality in carpal tunnel release. J Hand Surg Eur Vol. (2019) 44(3):278–82. 10.1177/1753193418808182 30394830

[30] Lye KW Waite PD Meara D Wang D. Quality of life evaluation of maxillomandibular advancement surgery for treatment of obstructive sleep apnea. J Oral Maxillofac Surg. (2008) 66(5):968–72. 10.1016/j.joms.2007.11.031 18423288

[31] Carrion MJ Nunes ML Martinez JV Portuguez MW da Costa JC. Evaluation of sleep quality in patients with refractory seizures who undergo epilepsy surgery. Epilepsy Behav. (2010) 17(1):120–3. 10.1016/j.yebeh.2009.11.008 20004148

[32] Muthukrishnan A Muralidharan TR Subash J Lathamangeswari C. Association of poor sleep quality with risk factors after coronary artery bypass graft surgery-a prospective cohort study. J Vasc Nurs. (2020) 38(2):83–92. 10.1016/j.jvn.2020.02.001 32534658

[33] Tsui WK Yang Y McGrath C Leung YY. Improvement in quality of life after skeletal advancement surgery in patients with moderate-to-severe obstructive sleep apnoea: a longitudinal study. Int J Oral Maxillofac Surg. (2020) 49(3):333–41. 10.1016/j.ijom.2019.07.007 31353172

[34] Nourizadeh N Rasoulian B Majidi MR Ardani AR Rezaeitalab F Asadpour H. Sleep quality after endoscopic Sinus surgery in patients with sinonasal polyposis. Auris Nasus Larynx. (2019) 46(6):866–70. 10.1016/j.anl.2019.03.002 30910416

[35] Jiang Z Zhou G Song Q Bao C Wang H Chen Z. Effect of intravenous oxycodone in combination with different doses of dexmedetomdine on sleep quality and visceral pain in patients after abdominal surgery: a randomized study. Clin J Pain. (2018) 34(12):1126–32. 10.1097/AJP.0000000000000645 30134283

[36] Li HJ Li CJ Wei XN Hu J Mu DL Wang DX. Dexmedetomidine in combination with morphine improves postoperative analgesia and sleep quality in elderly patients after open abdominal surgery: a pilot randomized control trial. PLoS One. (2018) 13(8):e0202008. 10.1371/journal.pone.0202008 30106963 PMC6091958

[37] Yasar NF Badak B Canik A Baş S Uslu S Öner S Effects of sleep quality on melatonin levels and inflammatory response after Major abdominal surgery in an intensive care unit. Molecules. (2017) 22(9):e1537. 10.3390/molecules22091537 28895895 PMC6151787

[38] Mansano-Schlosser TC Ceolim MF Valerio TD. Poor sleep quality, depression and hope before breast cancer surgery. Appl Nurs Res. (2017) 34:7–11. 10.1016/j.apnr.2016.11.010 28342628

